# Influence of Slurry Diluents on Toothpaste Efficacy Against Erosive Tooth Wear: *In Vitro* and *In Situ* Studies

**DOI:** 10.1590/0103-644020256448

**Published:** 2025-11-21

**Authors:** Laura Nobre Ferraz, Isabele Vieira, Gabriel Candolato de Almeida, Tatiane Cristina Dotta, Waldemir Francisco Vieira-Junior, Núbia Inocêncya Pavesi Pini, Gláucia Maria Bovi Ambrosano, Débora Alves Nunes Leite Lima

**Affiliations:** 1 Hermínio Ometto Foundation, University of Araras, Araras, São Paulo, Brazil.; 2 Department of Restorative Dentistry, Piracicaba Dental School, University of Campinas, Piracicaba, SP, Brazil; 3 Department of Chemistry, Ribeirão Preto Faculty of Philosophy, Sciences and Letters at Ribeirão Preto, University of São Paulo, Ribeirão Preto, Brazil; 4 Department of Restorative Dentistry, São Leopoldo Mandic Institute and Dental Research Center, Campinas, SP, Brazil; 5 Department of Prosthodontics and Restorative Dentistry, Uningá University Center, Maringá, PR, Brazil; 6 Department of Social Dentistry, Piracicaba Dental School, University of Campinas, Piracicaba, SP, Brazil

**Keywords:** tooth erosion, toothpastes, enamel

## Abstract

Different diluents are used to prepare toothpaste slurries *in vitro* and *in situ* erosive tooth wear studies. This study aimed to evaluate the influence of diluents on the anti-erosive potential of toothpastes with different active agents. Bovine enamel specimens were assigned to groups (n = 12) according to slurry type (*in vitro* deionized water, *in vitro* remineralizing solution, clarified human saliva, or *in situ* human saliva) and toothpaste formulation: placebo (0 ppm F), sodium monofluorophosphate (1450 ppm F - MFP), sodium fluoride (1450 ppm F - NaF), stannous fluoride + sodium fluoride (1100 ppm F SnF₂ + 350 ppm F NaF - NaF/SnF₂), or chitosan (0.5%) + sodium fluoride (700 ppm F) + amine fluoride (700 ppm F) - Chi/NaF/AmF. A 5-day erosion cycle was performed, intercalating four erosive challenges (1 min, 1% citric acid, pH 3.5) and two remineralizing treatments, applied before the first and after the last erosive challenge each day (brushing for 15 seconds and immersion in slurry for up to 2 minutes). Surface microhardness (SMH) and tissue loss by contact profilometry were measured, and percentage SMH loss (%SMH) was calculated. Across all toothpastes, *in situ* human saliva resulted in the highest SMH and lowest tissue loss. Under *in vitro* conditions, NaF/SnF₂ and Chi/NaF/AmF slurries with human saliva yielded the best outcomes. For other formulations, no significant differences were observed between *in vitro* diluents. Results indicate that the diluent type influences SMH and profilometry outcomes, depending on the active agents. Toothpaste containing Sn, chitosan, and fluoride showed the most significant anti-erosive potential.

## Resumo

Diferentes tipos de diluentes são utilizados para fazer suspensões de creme dental em experimentos de desgaste dentário erosivo *in vitro* e *in situ*. O objetivo deste estudo foi avaliar a influência de diferentes diluentes no potencial antierosivo de creme dentais com diferentes agentes ativos em experimentos de desgaste dentário erosivo. Espécimes de esmalte bovino foram divididos em grupos (n=12) de acordo com a suspensão (água deionizada *in vitro*, solução remineralizante *in vitro*, saliva humana clarificada *in vitro* ou saliva humana *in situ*) e creme dental (0 ppm de flúor - placebo; 1450 ppm de F como monofluorofosfato de sódio - MFP; 1450 ppm de F como fluoreto de sódio - NaF; 1100 ppm de F como fluoreto estanoso + 350 ppm de F como fluoreto de sódio - NaF/SnF2; e Quitosana 0,5%, + 700 ppm de F como fluoreto de sódio + 700 ppm de F como fluoreto de amina - Chi/NaF/AmF). Um ciclo de erosão de 5 dias foi realizado intercalando 4 desmineralizações (1 min, 1% de ácido cítrico, pH = 3,5) e 2 remineralizações antes do primeiro e após o último desafio erosivo de cada dia (escovação por 15s e imersão em lama por até 2 minutos de tratamento). A microdureza de superfície (SMH) e a perda de tecido por meio de perfilometria de contato foram realizadas. A perda de microdureza de superfície (%SMH) foi calculada. Para todos os cremes dentais, a saliva humana *in situ* apresentou a maior SMH e a menor perda de tecido. Em condições *in vitro*, a maior SMH e a menor perda de tecido foram observadas para NaF/SnF2 e Chi/NaF/AmF com saliva humana. Para os outros cremes dentais, não foram encontradas diferenças entre os diluentes usados *​​in vitro* para nenhuma das análises. Concluiu-se que os diluentes tiveram influência nos resultados de SMH e perfilometria de acordo com o agente ativo do creme dental. Sn com quitosana e flúor apresentou o melhor potencial antierosivo.

**Figure:**
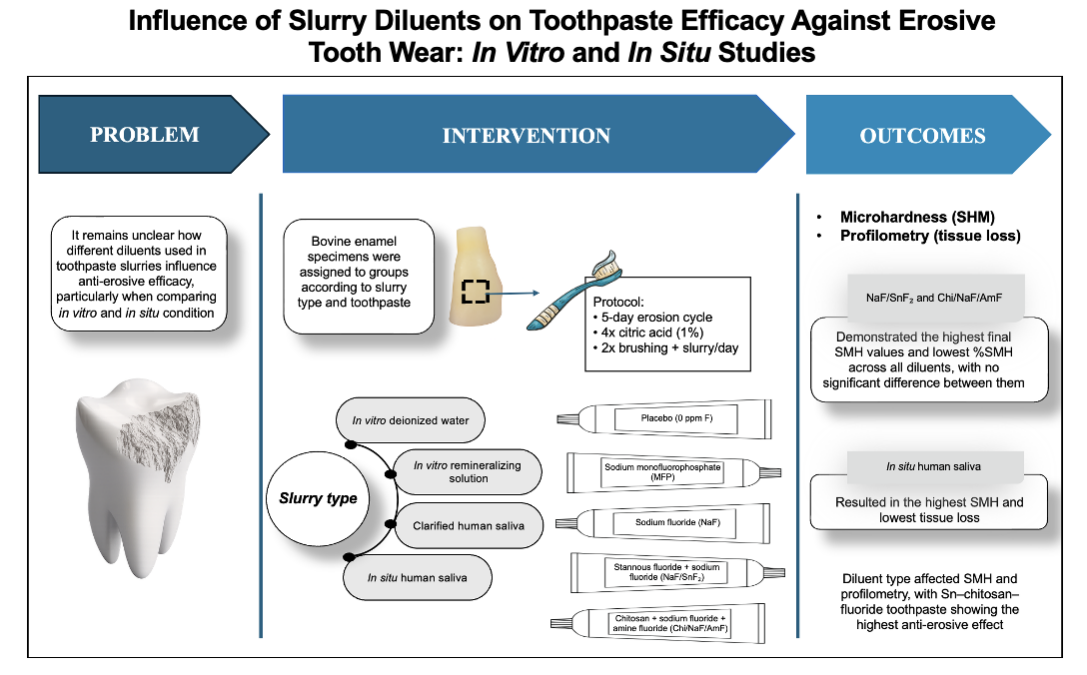

## Introduction

Several studies have been conducted to address the treatment and prevention of erosive tooth wear ^1^. Different study designs have been developed to better understand the role of toothpaste in mitigating this phenomenon ^1^. *In vitro* models are the most prevalent approach, as they afford greater control and standardization over several parameters ^2^. Additionally, these models provide a high level of scientific control, resulting in lower variability and require a smaller sample size ^3^. In comparison with *in situ* and *in vivo* studies, *in vitro* experiments on erosive tooth wear allow for the evaluation of isolated experimental variables over a comparatively short duration and with an advantageous cost-benefit profile ^2^. As the majority of research on erosive tooth wear has been conducted *in vitro*, it is crucial that these experiments accurately replicate clinical scenarios to ensure the scientific relevance of the results generated. One significant consideration in these studies pertains to the use of diluents to simulate clinical conditions and their potential impact on the prevention and treatment of erosive tooth wear.

Given that erosive tooth wear is a non-bacterial chemical process, *in vitro* and *in situ* investigations often focus on simulating both the erosive process and subsequent treatment ^4^. These studies frequently involve alternating cycles of dental tissue demineralization using acids (such as citric or hydrochloric acid) and mineral recovery with active agents ^5^. A significant area of research involves evaluating the efficacy of different fluoride compounds present in toothpaste, including sodium monofluorophosphate (MFP), sodium fluoride (NaF), and stannous fluoride (SnF_2_). In addition, novel agents, such as the biopolymer chitosan, are being explored for their potential to augment the anti-erosive effects of fluoride compounds ^6^. To establish dependable experimental conditions, models often simulate toothbrushing events, replicating the mechanical forces within the oral cavity. The dilution of toothpaste, which naturally occurs upon contact with saliva, is another crucial factor taken into consideration in testing. Consequently, *in vitro* models utilize various diluents for toothpaste slurries, including distilled water ^5^, artificial saliva/remineralizing solution ^7^, or processed natural human saliva ^8^, typically at a 1:3 weight ratio of toothpaste to diluent. This proportion is based on evidence in the literature indicating its prevalence during oral hygiene practices ^2^. It is important to note that the rheological properties of the diluents used in preparing the toothpaste slurry can impact toothbrushing abrasion ^9^. Moreover, the choice of diluent may influence the anti-erosive effectiveness of toothpaste, as certain active principles require salivary enzymes for their bioavailability ^3^.

This issue warrants significant attention as saliva plays a fundamental role in the mechanism of fluoride. The protective efficacy of fluoride and the formation of CaF_2_ layers are contingent upon the concentration and specific fluoridated compounds, the pH, and the availability of calcium and phosphate mineral ions present in toothpaste, as well as in saliva ^10^. Therefore, the presence of human saliva enables fluoride to deposit a greater quantity of CaF_2_ onto hard dental tissues, thereby contributing to the inhibition of demineralization and facilitating remineralization ^11^. Furthermore, saliva has the potential to interact with the action of chitosan. Speculation regarding this interaction suggests that the binding of chitosan to enamel and the proteins of the acquired pellicle, particularly mucin, may contribute to the reinforcement of the protective organic layer ^7^.

While diverse erosive protocols have been reported, the extent and manner in which human saliva or its substitutes affect these experimental models are not well-established. Moreover, there is a lack of comparative studies between *in vitro* and *in situ* findings that have employed different diluents in the context of erosive tooth wear research. Consequently, the objective of this study was to investigate the influence of different diluents used in the preparation of toothpaste slurries on the anti-erosive efficacy of various active agents in erosive tooth wear experiments. The null hypotheses were as follows: ^1^ For each type of toothpaste, there is no difference in efficacy against erosive tooth wear when different diluents are compared; ^2^ For each type of diluent, there is no difference in efficacy against erosive tooth wear when different toothpastes are compared.

## Materials and methods

### Experimental design

A two-factor experimental design was utilized in this study, examining the influence of two independent variables: diluent (at four levels: *in vitro* deionized water, *in vitro* remineralizing solution, *in vitro* clarified human saliva, and *in situ* human saliva) and toothpaste (at five levels: placebo, MFP, NaF, NaF/SnF_2_, and Chi/SnF_2_/NaF). Specific details about the toothpastes employed can be found in Table 1.

**Table 1:** Toothpaste used in the study.

Toothpaste	Manufacture	Active principle	Others componentes
Placebo	Drogal Manipulation	-	Glycerin, Silica, Carboximethyl cellulose, water, Methyl P, Saccharin, Titanium dioxide, Sodium Lauryl Sulfate, Mint oil
Colgate ® Máxima Proteção Anticáries (MFP)	Colgate-Palmolive, São Bernardo do Campo, Brazil	Sodium monofluorophosphate (MFP) 1450 ppm	Calcium Carbonate, Water, Glycerin, Sodium Lauryl Sulfate, Aroma, Cellulose Gum, Tetrasodium Pyrophosphate, Sodium Bicarbonate, Benzyl Alcohol, Sodium Saccharin, Sodium Hydroxide.
Colgate Total 12® (NaF)	Colgate-Palmolive, São Bernardo do Campo, Brazil	Sodium Fluoride (NaF) 1450 ppm	Water, Triclosan, Sorbitol, Hydrated Silica, Sodium Lauryl Sulfate, PVM / MA Copolymer, Flavor, Carrageenan, Sodium hydroxide, Sodium Saccharin, Titanium dioxide, and Dipentene.
Oral-B® Pró-saúde Gengivas Saudáveis (NaF/SnF_2_)	Protect & Gamble, Gross Geral, Germany	Stannous Fluoride (SnF2) 1100 ppm and Sodium Fluoride (NaF) 350 ppm.	Glycerin, Silica, Sodium Hexametaphosphate, Propylene Glycol, PEG-6, Water, Zinc Lactate, Aroma, Sodium Gluconate, Titanium Dioxide (CI 77891), Sodium Lauryl Sulfate, Sodium Saccharin, Carrageenan, Trisodium Phosphate, Chloride Tin, Xanthan Gum, Cinnamal.
Elmex® Erosion Protectionᵀᴹ (Chi/NaF/AmF)	GABA International AG, Grabetsmattweg, Switzerland	Amine Fluoride (AmF) 700 ppm, Sodium Fluoride (NaF) 700 ppm, and Chitosan (0.5%), Stannous Chloride	Glycerin.Hydrated, Silica, Sodium hexametaphosphate, Propylene glycol, PEG-6, Eau, Zinc lactate, trisodium phosphate, flavor, Sodium lauryl sulfate, Polyethylene, Sodium, Gluconate, Carrageenan, Sodium saccharin, Xanthan gum, Titanium dioxide, red 40 aluminium lake

### Volunteers and Ethical Aspects

This research was carried out in adherence to the principles outlined in the Declaration of Helsinki and was approved by the local ethics committee for research (process Nº: 55292116.3). Twelve volunteers (six males and six females), ranging in age from 23 to 35 years, were enrolled in the study following the provision of written informed consent. All participants satisfied the inclusion criteria (normal salivary flow rate, absence of dental caries and/or periodontal disease, healthy or sufficiently restored dentition, and adequate oral hygiene practices) and presented none of the exclusion criteria (use of orthodontic devices or prostheses, use of medications known to interfere with salivary flow, current smoking, pregnancy or lactation, allergies to oral hygiene products, dental materials, or therapeutic agents employed in the research). Comprehensive details regarding the study protocol were provided to all volunteers before they signed the Informed Consent Form. 

### Saliva collection

Prior to saliva collection, volunteers were instructed to refrain from any oral hygiene measures and not consume breakfast. Salivary flow was stimulated through the mastication of paraffin wax (Parafilm M, American National Can- Chicago, IL), and the elicited saliva was collected in Falcon tubes maintained within an ice-filled beaker. The saliva obtained from all volunteers was subsequently pooled. This pooled saliva underwent centrifugation (Jouan MR23i Benchtop High-Speed Centrifuge, Thermo Scientific, Waltham, MA, USA) at 3,800g for 10 minutes at 4°C ^5^. Following centrifugation, the precipitate was discarded, and the resulting supernatant was stored individually at -80°C in volumes appropriate for each day of experimentation ^5^. Saliva collection was performed over a period of five days to accumulate a total volume of 2,000 mL, which was required for the entire study. Daily, aliquots of clarified human saliva were thawed at room temperature and homogenized before being utilized for acquired pellicle formation and slurry preparation.

### Specimen preparation

Four hundred and eighty enamel/dentin specimens (4 x 4 mm) were obtained from bovine incisors for this study, with 240 specimens designated for the *in vitro* phase and 240 for the *in situ* phase (n=12 per condition). The specimens were sectioned using a precision saw (Isomet 1000; Buehler, IL, USA) equipped with a diamond cutting disc (Buehler, Illinois, USA). The enamel surfaces underwent sequential grinding under water cooling on a polishing machine (Arotec, São Paulo, SP, Brazil) using 600-, 1000-, and 2000-grit silicon carbide (SiC) papers (Buehler, Illinois, USA). This was followed by polishing with felts (TCT, TWI, FVC - Arotec, Cotia, SP, Brazil) in conjunction with diamond polishing pastes of decreasing particle sizes (6 μm, three μm, and ¼ μm - Buehler, IL, USA). The resulting enamel specimens had a standardized thickness of 1 mm for both the enamel and dentin portions. All specimens were subjected to chemical sterilization using ethylene oxide under wet conditions ^12^. To facilitate surface profilometry analysis, half of the surface area of each specimen was protected with an acid-resistant varnish, creating an untreated reference area alongside the test area.

### Intraoral appliances

Plaster casts (ASFER, São Caetano do Sul, SP, Brazil) were produced from alginate impressions (Jeltrate - Dentsply, Petrópolis, RJ, Brazil) obtained from the volunteers. The intraoral appliances were manufactured using acrylic resin (VIPI, Pirassununga, SP, Brazil) and were designed to replicate the individual palatal morphology of each volunteer, ensuring accurate placement in the palatal area. Four specimens were mounted somewhat inferior to the appliance surface. This positioning aimed to prevent any direct contact between the specimen surface and the tongue, while still allowing adequate access for toothbrushing.

### *In vitro* model

A 5-day erosion cycling model was applied ^5^. Prior to the initial erosive challenge of each day, the specimens were individually immersed in clarified human saliva at 37°C under agitation (100 rpm, 2.5 ml/mm² for exposed enamel) for a duration of 1 hour ^5^. Subsequently, they were subjected to immersion in a 1% citric acid solution, adjusted to pH 3.5, for 1 minute, also under agitation (100 rpm - 2.5 ml/mm² for exposed enamel), at room temperature. This erosive cycle was repeated four times daily, with a 1-hour interval between each immersion ^5^. The citric acid solution was prepared fresh daily and replenished for every erosion episode.

Before the first and after the final erosive challenge of each day, the specimens were immersed in their respective treatment slurries (2.5 ml/mm² for exposed enamel), prepared at a weight ratio of 1 part toothpaste (1.5g) to 3 parts diluent (4.5 ml). These slurries were prepared immediately before their application. Brushing of the specimens was performed using an electric toothbrush equipped with a pressure sensor ^13^ (Oral-B Professional Care 3000; Oral-B, Schwalbach am Taunus, Germany). To standardize the positioning and applied force of the toothbrush on the specimens, a custom-designed rigid silicone device was employed ^14^. This device ensured that the brush head remained parallel to the specimen surface throughout the entire brushing procedure ^14^. Furthermore, the device was engineered to exert a precise pressure of 2.5N, indicated by a visual light alert, when the brush head was positioned on the specimen.

The same model of brush head was used for all experimental groups, with a dedicated brush head assigned to each group to prevent any interaction between the different toothpastes and diluents. For the brushing process, the brush heads were secured to the custom device, and the operator maintained a firm grip on the brush body to ensure continuous contact with the device and prevent any unintended manual movement. The brush was activated for a duration of 15 seconds on each specimen, maintaining consistent pressure and without additional manual manipulation ^14^. Following brushing, the specimens remained immersed in the slurry for a total treatment time of 2 minutes. All brushing procedures were consistently performed by the same trained operator ^15^. Between the erosive challenges (1 hour) and after completing the daily cycle, the specimens were stored in a remineralizing solution at 37°C overnight (20 hours). The composition of the remineralizing solution was 1.5 mmol/L Ca, 0.9 mmol/L P, 150 mmol/L KCl, 0.1 mol/L Tris buffer, with a final pH of 7.0 ^16^.

### *In situ* model

The *in situ* phase of the study was carried out in 5 distinct five-day periods, separated by 7-day washout intervals. Each volunteer (n = 12) constituted a single experimental unit, and analyses were performed on four specimens per intraoral appliance to calculate the mean and derive a single representative value for each volunteer. Prior to the commencement of each experimental cycle and throughout the 5-day cycle, volunteers were instructed to use a fluoride-free dentifrice for a 3-day washout period to prevent any confounding effects on the treatments. The same cyclic model employed in the *in vitro* phase was also implemented *in situ*. The procedures requiring volunteer participation were the 1-hour in situ exposure of specimens to saliva before the first daily erosive challenge and the preparation of the treatment slurry. All other procedures, including erosion, brushing, and storage in remineralizing solution, were performed in the same manner as described for the *in vitro* phase. For specimen brushing, a standardized quantity of 1.5 g of toothpaste was used for each phase and participant. With the intraoral device positioned in the mouth, volunteers initially brushed the labial/buccal surfaces of their own teeth for 30 seconds to generate the toothpaste-saliva suspension ^12^. Following this, the specimens were maintained in contact with the slurry for 1 minute. The intraoral appliance was then removed, and each specimen underwent a 15-second brushing, resulting in a total treatment duration of 2 minutes.

### Surface microhardness and Surface microhardness loss

Surface microhardness (SMH) measurements were performed at baseline (SMH initial) and following the final erosive challenge (SMH final). A microhardness tester (HMV-2000 Shimadzu, Tokyo, Japan) equipped with a Knoop indenter was employed. A load of 50 g was applied for a dwell time of 5 seconds. Three indentations, with a 100 μm interval between each, were made in the central region of the enamel surface. The distance between indentations corresponding to different measurement time points was also maintained at 100 μm. The percentage of surface microhardness loss (%SMH) was calculated using the following formula: %SMH = 100 × [(SMH final - SMH initial) / SMH initial].

### Surface profilometry

Tissue loss was analyzed using profilometry (Veeco DEKTAK 150, Veeco, NY, USA) after the conclusion of the final experimental day. On each specimen, three parallel traces, separated by intervals of 0.2 mm and each extending 2 mm in length (1 mm on the reference surface and 1 mm on the experimental surface), were recorded across the central region. The minimum level of detection for the profilometer was 4 angstroms. For each trace, two regression lines were generated: one corresponding to the reference area and the other to the experimental area. The vertical displacement between these two regression lines was defined as the extent of tissue loss, expressed in micrometers (μm). The tissue loss value for each specimen was determined by calculating the arithmetic mean of the three measurements.

### Statistical analysis

Following the initial exploratory analysis, the microhardness data were subjected to analysis using mixed linear models for repeated measures over time. Where significant effects were observed, post-hoc comparisons were performed using the Tukey-Kramer test to identify specific differences between groups. Tissue loss data and surface microhardness loss data were analyzed using a two-way analysis of variance (ANOVA). Significant main effects and interactions were further investigated using the Tukey post-hoc test. All statistical analyses were performed using SAS software, with a significance level of 5%.

## Results

### Surface microhardness and surface microhardness loss

The results of surface microhardness (SMH) and surface microhardness loss (%SMH) are presented in Tables 2 and 3.

**Table 2:** Average (standard deviation) of surface microhardness analysis by group and time (n = 12).

Time	Toothpaste	Diluent			
		*in vitro* deionized water	*in vitro* remineralizing solution	*in vitro* clarified human saliva	*in situ* human saliva
Initial	Placebo	*323.9 (11.0) Aa	*323.8 (12.2) Aa	*323.4 (12.7) Aa	*331.4 (9.3) Aa
	MFP	*322.8 (11.6) Aa	*323.7 (12.2) Aa	*324.2 (11.5) Aa	*330.9 (9.4) Aa
	NaF	*324.1 (12.8) Aa	*323.5 (12.2) Aa	*324.0 (12.0) Aa	*332.2 (9.5) Aa
	NaF/SnF_2_	*324.3 (12.5) Aa	*323.1 (12.1) Aa	*323.1 (13.3) Aa	*330.0 (10.4) Aa
	Chi/NaF/AmF	*323.6 (11.7) Aa	*323.3 (12.9) Aa	*323.0 (12.9) Aa	*330.9 (10.6) Aa
Final	Placebo	173.0 (8.3) Bc	176.6 (6.9) Bc	170.5 (8.1) Bc	201.1 (7.3) Ad
	MFP	172.8 (7.9) Bc	175.4 (8.2) Bc	176.8 (9.7) Bc	224.0 (6.1) Ac
	NaF	210.9 (7.3) Bb	210.2 (10.4) Bb	205.5 (11.6) Bb	272.9 (7.2) Ab
	NaF/SnF_2_	241.6 (11.2) Ba	246.2 (6.0) Ba	273.6 (8.2) Aa	289.3 (9.6) Aa
	Chi/NaF/AmF	238.3 (5.9) Ca	250.9 (9.0) Ca	275.1 (6.8) Ba	300.3 (6.6) Aa

*Statistically different from the final time (p≤0.05). Means followed by distinct letters (uppercase in the horizontal and lowercase in the vertical, comparing toothpaste within each time) differ from each other (p≤0.05). p(toothpaste)<0.0001; p(diluent)= 0.0031; p(toothpaste x diluent)= 0.0309.

All groups exhibited a significant reduction in SMH between the initial and final measurements, regardless of the toothpaste or diluent used. Notably, an inverse relationship was observed between SMH and %SMH: groups with higher final SMH values exhibited lower percentage loss, while groups with lower final SMH showed greater loss.

**Table 3:** Average (standard deviation) of surface microhardness loss (%) by toothpaste and diluent (n=12).

Toothpaste	Diluent			
	*in vitro* deionized water	*in vitro* remineralizing solution	*in vitro* clarified human saliva	*in situ* human saliva
Placebo	46.6 (2.5) Aa	45.3 (3.7) Aa	47.2 (2.7) Aa	39.3 (3.0) Ba
MFP	46.4 (2.4) Aa	45.7 (3,5) Aa	45.4 (4.2) Aa	32.2 (3.1) Bb
NaF	34.8 (3.7) Ab	34.9 (3.9) Ab	36.5 (4.4) Ab	17.8 (3.1) Bc
NaF/SnF_2_	25.4 (3.8) Ac	23.7 (2.9) Ac	15.2 (4.2) Bc	12.3 (3.5) Bd
Chi/NaF/AmF	26.2 (3.8) Ac	22.3 (3.2) Ac	14.7 (4.1) Bc	9.1 (4.5) Cd

Means followed by different letters (uppercase in the horizontal and lowercase in the vertical) differ from each other (p≤0.05). p(toothpaste)<0.0001; p(diluent<0,0001; p(toothpaste x diluent)<0,0001.

Across the in vitro deionized water, remineralizing solution, and clarified human saliva conditions, the placebo and MFP toothpastes consistently showed the lowest final SMH and the highest percentage of SMH, with no statistically significant differences between them. In the in situ human saliva condition, MFP toothpaste differed from the placebo but maintained the same trend.

NaF/SnF2 and Chi/NaF/AmF toothpastes demonstrated the highest final SMH values and the lowest percentage of SMH across all diluents, with no significant difference between them. NaF toothpaste showed intermediate outcomes, differing significantly from the other groups depending on the diluent.

Regarding the influence of diluents, in situ human saliva provided the most protective effect for placebo, MFP, and NaF toothpastes, resulting in higher final SMH and lower percentage of SMH compared to in vitro conditions. For NaF/SnF2 and Chi/NaF/AmF, both in vitro and in situ human saliva produced similarly favorable results.

### Surface profilometry

The outcomes of the tissue loss analysis, as determined by profilometry, are detailed in Table 4.

**Table 4:** Mean (standard deviation) of surface profilometry analysis according to the group (n=12).

Toothpaste	Diluent			
	*in vitro* deionized water	*in vitro* remineralizing solution	*in vitro* clarified human saliva	*in situ* human saliva
Placebo	2.6 (0.4) Ba	2.6 (0.4) Ba	2.7 (0.4) Ba	2.6 (0.3) Ba
MFP	2.7 (0.4) Ba	2.7 (0.4) Ba	2.6 (0.3) Ba	2.4 (0.3) Ba
NaF	1.8 (0.2) Bb	1.8 (0.2) Bb	1.8 (0.2) Bb	1.8 (0.2) Bb
NaF/SnF_2_	1.1 (0.1) Bc	1.0 (0.2) Bc	1.1 (0.3) Bc	0.5 (0.2) Ac
Chi/NaF/AmF	1.0 (0.3) Bc	1.0 (0.2) Bc	1.0 (0.2) Bc	0.5 (0.2) Ac

Means followed by different letters (uppercase in the horizontal and lowercase in the vertical) differ from each other (p≤0.05).

For all the diluents evaluated, the placebo and MFP groups showed the highest values and did not differ statistically from each other. The NaF group showed intermediate values and differed from the other groups. The NaF/SnF_2_ and Chi/NaF/AmF groups showed the lowest values, which did not differ between them, but were significantly different from all the other groups.

For placebo, MFP, and NaF toothpaste, no statistically significant differences were found between the diluents. For NaF/SnF_2_ and Chi/NaF/AmF toothpaste, the *in situ* human saliva showed the highest values ​​and differed statistically from all the other diluents. The *in vitro* deionized water, *in vitro* remineralizing solution, and *in vitro* human saliva showed the lowest values ​​and did not differ among them.

## Discussion

This study investigated the influence of different diluents used in slurry preparation on the anti-erosive potential of various toothpastes in an erosive tooth wear model. Because toothpastes were applied with abrasion, their abrasive potential was considered relevant. Relative dentin abrasivity (RDA) and relative enamel abrasivity (REA) are commonly used to assess toothpaste abrasivity ^17^. According to ADA recommendations, toothpastes should have RDA values below 250 to be considered safe when used with appropriate force and a toothbrush. Although abrasion may increase with higher RDA values ^18^, this relationship is not fully established ^19^. Most oral-care products on the market have been tested only for their RDA, but not for their REA; therefore, RDA is commonly used to describe toothpaste. ^20^. As noted by previous authors ^17^, RDA is one of many factors to consider in managing erosive tooth wear, and this study also investigated other relevant variables.

The first hypothesis, which stated that the diluent would not significantly influence the anti-erosive efficacy of the tested toothpastes, was rejected. Surface microhardness analysis revealed that the diluent had a significant impact on the anti-erosive potential of all toothpastes evaluated. Similarly, profilometry analysis showed that the diluents interfered with the anti-erosive effect of the NaF/SnF2 and Chi/NaF/AmF toothpastes. The second hypothesis, which proposed no statistically significant differences in anti-erosive potential among the toothpastes across all diluents, was also rejected. The results revealed statistically significant variations in anti-erosive efficacy according to the active agent composition of the toothpastes for all diluents tested.

The hypotheses of this study were tested using surface profilometry and surface microhardness analyses. Surface profilometry provides a quantitative assessment of dental tissue loss relative to an untreated reference area ^21^ and is frequently applied in numerous *in vitro* ^5^ and *in situ* studies ^22^. While profilometry can measure tissue loss resulting from erosion, abrasion, or their combination, it does not provide information on the depth of enamel surface softening ^21^. Surface softening, defined as a decrease in hardness, is assessed by evaluating the substrate's resistance to the penetration of an indenter ^21^. The Knoop diamond penetrates sound enamel by approximately 1.5 µm under the usual 50 g load ^21^ ^,^ ^23^. Alterations in surface enamel hardness can be observed even after short periods of exposure to an erosive agent ^24^, rendering microhardness analysis a simple yet accurate method for detecting early-stage erosion ^21^.

Given the aim of this study - to evaluate the effect of different slurry types associated with toothpastes - both surface microhardness and profilometry were employed. Profilometry quantified structural loss, while microhardness assessed changes in the softened surface layer that can persist even under repeated acid challenges. This dual approach was important because no previous studies have examined this specific combination of slurries and toothpastes, and it was uncertain whether the tested conditions would lead to early erosion or to erosive tooth wear. This uncertainty was mainly attributed to the presence of human saliva, as the in situ pellicle significantly reduces toothpaste abrasion ^25^ and saliva lubricates the tooth surface ^26^ ^,^ ^27^ ^,^ ^28^, potentially decreasing abrasion ^25^. Furthermore, previous studies have successfully utilized microhardness in erosive models, reporting significant differences among experimental groups ^5^ ^,^ ^15^ ^,^ ^29^ ^,^ ^30^ ^,^ ^31^ ^,31)^, which may reflect variations in mineral loss patterns or rehardening effects following exposure to toothpaste and saliva. In light of these considerations, the inclusion of both analytical methods was deemed essential.

Under in situ conditions, the presence of human saliva during placebo toothpaste application likely influenced surface microhardness outcomes. This group demonstrated the lowest microhardness values and the most significant surface microhardness loss when compared to all other toothpastes across all tested diluents. Comparisons across diluents for the placebo toothpaste revealed higher microhardness and reduced surface microhardness loss, with significant differences when human saliva was employed under *in situ* conditions. This effect may reflect the role of saliva in modulating erosion, protecting multiple mechanisms. Saliva is rich in calcium and phosphate ions ^32^, which can reprecipitate at pH above 5.5, promoting remineralization ^32^.

The microhardness outcomes and surface microhardness loss in the placebo group under *in situ* human saliva may be attributed to saliva exposure and acquired pellicle formation. The acquired pellicle protects enamel by acting as a diffusion barrier, reducing phosphate and calcium ion loss under acidic conditions ^33^. *In situ*, the pellicle formed directly in volunteers' mouths, unlike other diluents, where specimens were immersed in collected human saliva. Its protective efficacy depends on physical properties ^34^. Saliva can alter collection, storage, and cycling, which in turn affect its protective effect ^35^. Protein breakdown and pH shifts may affect pellicle formation and function ^36^ ^,^ ^34^. These factors may explain differences between in situ and other diluent tests for the placebo toothpaste.

The lower anti-erosion potential of the placebo toothpaste compared to other toothpastes, regardless of diluent, may be attributed to the absence of fluoride or remineralizing components. Fluoride is widely used to treat and prevent dental erosion ^1^ ^,^ ^6^, promoting rehardening of softened tissues and reducing demineralization, thereby limiting tissue loss ^5^. Fluoride toothpastes and other remineralizing agents are routinely evaluated for anti-erosive potential and clinical applicability in dental erosion management ^1^. A fluoride toothpaste was included in this study to compare its active agents with those in literature protocols. The results indicated that the diluent also influenced the anti-erosive efficacy of the fluoride toothpaste. For all toothpastes, the highest microhardness and lowest loss occurred under in situ human saliva. Beyond remineralization during brushing, saliva influences CaF2 layer formation, as calcium and phosphate ions are essential ^10^. Additionally, fluoride in saliva generates more CaF2, supporting remineralization and preventing demineralization ^11^. Moreover, although all groups were positively influenced by in situ human saliva, the efficacy of each toothpaste against erosive tooth wear varied according to the type of fluoridated and other remineralizing agents present in their composition.

The diluent had a significant impact on the anti-erosive potential of the MFP-based toothpaste. Notably, *in situ* human saliva yielded higher microhardness values for the MFP toothpaste when compared to other diluents used with this formulation. Despite that, this toothpaste also demonstrated the most significant loss of surface microhardness. This observation may be attributed to the mechanism of action of MFP, which necessitates a hydrolysis step catalyzed by salivary enzymes to release fluoride ions, the active agents in remineralization. This enzymatic hydrolysis can diminish the effectiveness of MFP-containing toothpaste in *in vitro* investigations ^37^. Moreover, the influence of the *in situ* human saliva diluent on MFP resulted in a discernible difference between the performance of MFP and placebo toothpastes in terms of surface microhardness and tissue loss. However, it is crucial to note that MFP consistently exhibited the poorest performance across all diluents and throughout all analyses conducted. This finding corroborates the existing literature, which highlights the limited anti-erosive capacity of toothpaste formulated with MFP in the context of erosive tooth wear ^5^. The *in vitro* clarified human saliva diluent failed to enhance the efficacy of the MFP toothpaste, showing no significant difference from the placebo or other *in vitro* diluents evaluated. This lack of enhanced performance, despite the presence of human saliva during brushing, may be related to potential degradation of the saliva, which could impair the hydrolysis of MFP and consequently its anti-erosive potential against erosive tooth wear ^35^. Furthermore, the inherent instability of MFP within the toothpaste matrix, leading to the release of fluoride ions during storage and their subsequent reaction with calcium to form insoluble fluoride ^38^, may also contribute to these findings.

The diluent also influenced the anti-erosive efficacy of the NaF-based toothpaste. Regarding microhardness, *in situ* human saliva produced significantly higher values compared to the other tested diluents. Moreover, this toothpaste exhibited the lowest surface microhardness loss when *in situ* human saliva was used. Previous studies indicate that the effectiveness of NaF is modulated by saliva ^15^, with saliva concentration appearing to be a critical factor in its anti-erosive capacity ^15^. This may explain the absence of statistically significant differences between *in vitro* clarified human saliva and other *in vitro* diluents in terms of microhardness and surface microhardness loss. Despite variations among the diluents, NaF toothpaste exhibited comparable anti-erosive potential across all diluents relative to the other evaluated toothpastes, as measured by both microhardness and profilometry, with its effects being most apparent in comparison to the placebo and MFP toothpastes. The literature also supports a lower anti-erosive potential for NaF compared to other active ingredients ^5^ ^,^ ^14^ ^,^ ^15^. Under acidic conditions, NaF’s capacity to form a CaF2 layer on demineralized surfaces is limited due to the low pH ^10^.

The anti-erosive potential of stannous-containing toothpaste was affected by the diluent employed. Regarding microhardness, both *in vitro* clarified human saliva and *in situ* human saliva produced higher values, with no significant difference between them. In contrast, differences were observed when compared to the other diluents for the NaF/SnF2 toothpaste. For profilometry, in situ human saliva yielded lower values and differed from the other evaluated diluents. These findings align with previous studies ^15^, which reported that in the presence of saliva, stannous ions interact not only with mineralized tissues but also with proteins of the acquired pellicle ^39^, including mucins and albumins ^40^. Such interactions can increase pellicle thickness ^40^, forming a more substantial barrier against acids ^41^, which likely contributed to the higher values observed under human saliva conditions, as the same interaction did not occur with the other diluents evaluated in this study.

Although differences were observed among the diluents for stannous fluoride, toothpastes containing both fluoride and stannous ions demonstrated the highest anti-erosive efficacy in the analyses performed. Both NaF/SnF₂ and Chi/NaF/AmF exhibited the highest microhardness values and the lowest profilometry readings, independent of the diluent employed. These toothpastes also exhibited the lowest surface microhardness loss values and contained stannous in their formulation. Stannous-containing products have consistently demonstrated notable effectiveness in the remineralization of eroded enamel ^5^. SnF₂ can interact with and become incorporated into eroded enamel ^42^, forming salts with calcium and phosphate ^41^. Furthermore, the favorable performance of these toothpastes, even when combined with artificial saliva or distilled water as diluents, may be explained by the capacity of Sn²⁺ ions to react with mineral components in artificial saliva, such as calcium and phosphate, forming stannous-based precipitates on the tooth surface ^43^ ^,^ ^44^. In such cases, adsorption of Sn²⁺ ions is less efficient, and the resulting protective layer may be thinner or less stable compared to that formed in the oral environment. Nevertheless, even in the absence of proteins, the presence of calcium and phosphate ions in artificial saliva is sufficient to provide a partial protective effect of stannous fluoride ^43^ ^,^ ^44^ ^,^ ^45^.

The Chi/NaF/AmF toothpaste, in addition to ion stannous (Stannous Chloride), also contained chitosan in its formulation. Toothpastes combining chitosan, fluoride, and stannous have been reported to exhibit both anti-erosive and combined anti-erosive/anti-abrasive properties ^5^ ^,^ ^7^. In the present study, this toothpaste demonstrated favorable performance across all analyses conducted. Chitosan, a cationic biopolymer, can bind with proteins ^46^, fluoride ions ^47^, other ions present in saliva and on the enamel substrate ^6^, and abrasive particles within toothpaste formulations ^48^. The binding of chitosan to the dental surface facilitates the formation of protective layers, which enhance the retention of stannous ions on enamel ^49^, thereby promoting remineralization. Moreover, the chitosan layer can reduce frictional forces between tooth enamel and toothpaste abrasives ^50^, potentially decreasing tissue loss. The interaction between chitosan and saliva likely contributed to the observed variations among the diluents. For both surface microhardness and profilometry assessments, this toothpaste produced the most favorable values when in situ human saliva was used as the diluent, showing statistically significant differences from the other evaluated diluents. Additionally, in the presence of in vitro clarified human saliva, this toothpaste was unique in displaying higher values and differed from other in vitro diluents under investigation.

It is important to consider that, although the superior efficacy of stannous fluoride compared to sodium fluoride is well-documented, interactions with other formulation components, such as zinc and polyphosphates, may modulate remineralization and overall protective performance. Additionally, salivary constituents, particularly mucins, can interfere with tin ion retention on enamel surfaces, potentially reducing the protective efficacy of stannous fluoride (Hannig & Balz, 2001; Shellis & Addy, 2014). Interactions between different polymers, such as chitosan and mucin, may not always be synergistic, as competition for enamel binding sites can limit the deposition of protective layers and impact clinical effectiveness. Furthermore, several factors-both experimental (e.g., absence of dynamic salivary flow, use of static models) and biological (e.g., variations in salivary composition, diet, and oral hygiene practices)-may influence the actual *in vivo* performance of toothpastes.

Upon evaluating all results and comparing the findings from the two analytical methods employed in this study, it was observed that, for surface profilometry, statistically significant differences among diluents were evident only for the NaF/SnF2 and Chi/NaF/AmF toothpastes, with *in situ* human saliva associated with reduced tissue loss. Regarding surface microhardness, the presence of human saliva resulted in variations among the diluents when used *in vitro* for several types of toothpaste and *in situ* for all tested toothpastes. These findings suggest that, while the presence of saliva during brushing is relevant for microhardness, the abrasive properties of the toothpaste formulation and the mechanical action of toothbrush bristles appear to exert a greater influence on enamel wear than saliva alone. This phenomenon may be attributed to the fact that toothpastes act through chemical reactivity or mineral precipitation, mechanisms that are inherently dependent on an aqueous medium, regardless of its specific composition (i.e., with or without saliva).

Although not extensively addressed, the diluent can influence the action of toothpastes against erosive tooth wear. However, regarding profilometry, this impact appears minimal, as the presence of a diluent similar to water is generally sufficient. In contrast, for surface microhardness, the diluent is a significant factor that can impact the anti-erosive potential of toothpastes, depending on their active ingredients. The findings of this study should be considered in future investigations, as they may influence the evaluation of toothpaste efficacy. Understanding the chemical reactivity of toothpastes remains essential for determining their protective effect. While the use of saliva provides a model closer to clinical conditions, further studies comparing in vitro and in situ models are warranted to obtain results more representative of the oral environment. Additionally, future research could explore the relationships between RDA, REA, slurry composition, and the role of saliva as a diluent.

*In vitro* and *in situ* studies on erosive tooth wear should employ methodologies that ensure reliable results and closely reflect clinical conditions. This study demonstrated that the anti-erosive potential of toothpastes with different active ingredients varies according to the in vitro and in situ methodology applied, which may lead to outcomes that do not fully represent clinical reality. In microhardness analyses, the diluents influenced the results for all evaluated toothpastes, indicating that in vitro findings should be interpreted with caution before extrapolating to clinical settings. For surface profilometry, the diluent affected only toothpastes containing NaF/SnF₂ and Chi/NaF/AmF, suggesting that for other formulations, mineral loss *in vitro* was comparable to that observed *in situ*. The MFP-containing toothpaste demonstrated limited efficacy against erosive tooth wear.

In contrast, toothpastes containing stannous fluoride, combined with chitosan and other fluorides, exhibited the best performance and anti-erosive effects among the formulations tested. Despite these findings, further studies are necessary to assess the impact of various salivary constituents and polymer combinations under conditions more closely resembling the oral environment. Future research should also consider personalized approaches accounting for individual variability in pellicle formation and composition to optimize the preventive potential of oral care products.

## References

[1] Zanatta RF, Caneppele TMF, Scaramucci T, El Dib R, Maia LC, Ferreira DMTP (2020). Protective effect of fluorides on erosion and erosion/abrasion in enamel: a systematic review and meta-analysis of randomized in situ trials. Arch Oral Biol.

[2] Hara AT, Gonzµlez-Cabezas C, Creeth J, Zero DT (2008). The Effect of Human Saliva Substitutes in an Erosion-Abrasion Cycling Model. Eur J Oral Sci.

[3] Buzalaf MAR, Hannas AR, Magalhães AC, Rios D, Honório HM, Delbem ACB (2010). pH-cycling models for in vitro evaluation of the efficacy of fluoridated dentifrices for caries control: strengths and limitations. J Appl Oral Sci.

[4] Magalhães AC, Wiegand A, Buzalaf MAR (2014). Use of dentifrices to prevent erosive tooth wear: Harmful or helpful?. Braz Oral Res.

[5] Ferraz LN, Pini NIP, Ambrosano GMB, Aguiar FHB, Lima DANL (2019). Influence of cigarette smoke combined with different toothpastes on enamel erosion. Braz Oral Res.

[6] Lussi A, Carvalho TS (2015). The future of fluorides and other protective agents in erosion prevention. Caries Res.

[7] Carvalho TS, Lussi A (2014). Combined effect of a fluoride-, stannous- and chitosan-containing toothpaste and stannous-containing rinse on the prevention of initial enamel erosion-abrasion. J Dent.

[8] Lopes RM, da Silva JSA, João-Souza SH, Maximiano V, Machado AC, Scaramucci T (2020). Enamel surface loss after erosive and abrasive cycling with different periods of immersion in human saliva. Arch Oral Biol.

[9] Turssi CP, Messias DCF, Takeo Hara A, Hughes N (2010). Brushing abrasion of dentin: Effect of diluent and dilution rate of toothpaste.

[10] Moretto MJ, Magalhães AC, Sassaki KT, Delbem ACB, Martinhon CCR (2010). Effect of different fluoride concentrations of experimental dentifrices on enamel erosion and abrasion. Caries Res.

[11] Ganss C, Klimek J, Brune V, Schürmann A (2004). Effects of two fluoridation measures on erosion progression in human enamel and dentine in situ. Caries Res.

[12] Hara AT, González-Cabezas C, Creeth J, Parmar M, Eckert GJ, Zero DT (2009). Interplay between fluoride and abrasivity of dentifrices on dental erosion-abrasion. J Dent.

[13] Schlueter N, Klimek J, Ganss C. (2014). Effect of a chitosan additive to a Sn2+-containing toothpaste on its anti-erosive/anti-abrasive efficacy-a controlled randomised in situ trial. Clin Oral Investig.

[14] Comar LP, Gomes MF, Ito N, Salomão PA, Grizzo LT, Magalhães A C (2012). Effect of NaF, SnF 2, and TiF 4 toothpastes on bovine enamel and dentin erosion-abrasion in vitro. Int J Dent.

[15] Pini NIP, Schlueter N, Sundfeld D, Semper Hogg W, Santos-Silva AR, Lopes MA (2017). Efficacy of Stannous Ions on Enamel Demineralization under Normal and Hyposalivatory Conditions: A Controlled Randomized in situ Pilot Trial. Caries Res.

[16] Queiroz CS, Hara AT, Paes Leme AF, Cury JA (2008). pH-cycling models to evaluate the effect of low flouride dentifrices on enamel de-and remineralization. Braz Dent J.

[17] González-Cabezas C, Hara AT, Hefferren J, Lippert F (2013). Abrasivity testing of dentifrices - Challenges and current state of the art. Monogr Oral Sci.

[18] Wiegand A, Kuhn M, Sener B, Roos M, Attin T (2009). Abrasion of eroded dentin caused by toothpaste slurries of different abrasivity and toothbrushes of different filament diameter. J Dent.

[19] Hamza B, Niedzwiecki M, Körner P, Attin T, Wegehaupt FJ (2022). Effect of the toothbrush tuft arrangement and bristle stiffness on the abrasive dentin wear. Sci Rep.

[208] Hamza B, Attin T, Cucuzza C, Gubler A, Wegehaupt FJ (2020). RDA and REA Values of Commercially Available Toothpastes Utilising Diamond Powder and Traditional Abrasives. Oral Health Prev Dent.

[21] Schlueter N, Hara A, Shellis RP, Ganss C (2011). Methods for the measurement and characterization of erosion in enamel and dentine. Caries Res.

[22] Sakae LO, Niemeyer SH, Bezerra SJC, Borges AB, Turssi CP, Scaramucci T (2020). The Addition of Propylene Glycol Alginate to a Fluoride Solution to Control Enamel Wear: An in situ Study. Caries Res.

[23] Featherstone IJDE (1992). Consensus Conference on Intra-oral Models: Evaluation Techniques. J Dent Res.

[24] Hara AT, Zero DT (2008). Analysis of the erosive potential of calcium-containing acidic beverages. Eur J Oral Sci.

[25] Joiner A, Schwarz A, Philpotts CJ, Cox TF, Huber K, Hannig M (2008). The protective nature of pellicle towards toothpaste abrasion on enamel and dentine. J Dent.

[26] Lambrechts P, Debels E, Van Landuyt K, Peumans M, Van Meerbeek B (2006). How to simulate wear?. Overview of existing methods. Dental Materials.

[27] Christersson C, Lindh L, Arnebrant T (2000). Film-forming properties and viscosities of saliva substitutes and human whole saliva. Eur J Oral Sci.

[28] Berg C, Lindh L, Arnebrant T (2004). Intraoral lubrication of PRP-1, statherin and mucin as studied by AFM. Biofouling.

[29] Behl M, Taneja S, Bhalla VK (2024). Comparative evaluation of remineralization potential of novel bioactive agents on eroded enamel lesions: A single-blinded in vitro study. Journal of Conservative Dentistry and Endodontics.

[30] Boteon AP, dos Santos NM, Lamana LDBK, Rosa IMB, Di Leone CCL, Caracho RA (2023). Erosion-inhibiting and enamel rehardening effects of different types of saliva. Arch Oral Biol.

[31] Almansour A, Bartlett D, Addison O (2024). Impact of citric acid exposures on the erosion susceptibility and microhardness of anatomically different enamel surfaces. Dental Materials.

[32] Li X, Wang J, Joiner A, Chang J (2014). The remineralisation of enamel: A review of the literature. J Dent.

[33] Siqueira WL, Margolis HC, Helmerhorst EJ, Mendes FM, Oppenheim FG (2010). Evidence of intact histatins in the in vivo acquired enamel pellicle. J Dent Res.

[34] Vukosavljevic D, Custodio W, Buzalaf MAR, Hara AT, Siqueira WL (2014). Acquired pellicle as a modulator for dental erosion. Arch Oral Biol.

[35] Hall AF, Buchanan CA, Millett DT, Creanor SL, Strang R, Foye RH (1999). The effect of saliva on enamel and dentine erosion. J Dent.

[36] Batista GR, Torres CRG, Sener B, Attin T, Wiegand A (2016). Artificial saliva formulations versus human saliva pretreatment in dental erosion experiments. Caries Res.

[37] Ekambaram M, Itthagarun A, King NM (2011). Comparison of the remineralizing potential of child formula dentifrices. Int J Paediatr Dent.

[38] Cury JA, Tenuta LMA (2014). Evidence-based recommendation on toothpaste use. Braz Oral Res.

[39] Bellamy PG, Harris R, Date RF, Mussett AJS, Manly A, Barker ML (2014). In situ clinical evaluation of a stabilised, stannous fluoride dentifrice. Int Dent J.

[40] Algarni AA, Mussi MCM, Moffa EB, Lippert F, Zero DT, Siqueira WL (2015). The impact of stannous, fluoride ions and its combination on enamel pellicle proteome and dental erosion prevention. PLoS One.

[41] Schlueter N, Klimek J, Ganss C (2013). Randomised in situ study on the efficacy of a tin/chitosan toothpaste on erosive-abrasive enamel loss. Caries Res.

[42] Schlueter N, Hardt M, Lussi A, Engelmann F, Klimek J, Ganss C (2009). Tin-containing fluoride solutions as anti-erosive agents in enamel: an in vitro tin-uptake, tissue-loss, and scanning electron micrograph study. Eur J Oral Sci.

[43] Baig AA, Faller R V., Yan J, Ji N, Lawless M, Eversole SL (2014). Protective effects of SnF2 - Part I. Mineral solubilisation studies on powdered apatite. Int Dent J.

[44] Khambe D, Eversole SL, Mills T, Faller R V (2014). Protective effects of SnF2 - Part II. Deposition and retention on pellicle-coated enamel. Int Dent J..

[45] Kensche A, Buschbeck E, König B, Koch M, Kirsch J, Hannig C (2019). Effect of fluoride mouthrinses and stannous ions on the erosion protective properties of the in situ pellicle. Sci Rep.

[46] Van Der Mei HC, Engels E, De Vries J, Dijkstra RJB, Busscher HJ (2007). Chitosan adsorption to salivary pellicles. Eur J Oral Sci.

[46] Keegan GM, Smart JD, Ingram MJ, Barnes LM, Burnett GR, Rees GD (2012). Chitosan microparticles for the controlled delivery of fluoride. J Dent.

[48] Lundin M, Macakova L, Dedinaite A, Claesson P (2008). Interactions between chitosan and SDS at a low-charged silica substrate compared to interactions in the bulk - The effect of ionic strength. Langmuir.

[49] Hove LH, Holme B, Young A, Tveit AB (2008). The protective effect of tif4, SnF2 and NaF against erosion-like lesions in situ. Caries Res.

[50] Raviv U, Giasson S, Kampf N, Gohy JF, Jéröme R, Klein J (2003). Lubrication by charged polymers. Nature.

